# Gynecology and Obstetrics has Entered Modern Times: Perspectives and Challenges

**DOI:** 10.3389/fsurg.2014.00019

**Published:** 2014-06-03

**Authors:** Jean Bouquet de la Jolinière, Anis Fadhlaoui, Jean-Bernard Dubuisson, Anis Feki

**Affiliations:** ^1^Service de Gynécologie Obstétrique, HFR Fribourg – Hôpital Cantonal, Fribourg, Switzerland

**Keywords:** gynecology, obstetrics, reproductive techniques, assisted, laparoscopic surgery, genetics

The scientific progresses achieved, during the twenty-first century, in molecular biology, in cytogenetics, in the use of stem cells in the treatment of various pathologies by reprograming them (1), in medical imaging and in surgical instrumentation allow an avant-gardist management in the fields of assisted reproductive medicine, in oncology, in gynecological surgery, and even in obstetrics. Therefore, couples can benefit from a specific and personalized management. This management is supervised by multidisciplinary teams respecting laws of medical ethics and those of bio-ethics. Should we still use the term of “sterility” when such a therapeutic armada is at our disposal?

Born in the 70s, the laparoscopic surgery, now offers a better topographic approach of lesions, an almost perfect macroscopic depiction of colors. Hence, surgeons can spare more healthy tissues such as vessels and nerves, and thus adapt a more conservative procedure while restoring normal anatomy that is essential for preserving fertility. Endometriosis is one of the most obvious examples (2, 3). These advancements were achieved owing to new 3D imaging technologies and to the use of CO_2_ laser. The advent of new energies, i.e., RF, YAG laser, Argon laser, plasma energy, excimer, etc., opened new perspectives for surgical management of diseases.

The miniaturization of instruments in hysteroscopy coupled with the development of hydrosonography allowed a better exploration of the uterine cavity, endometrial biopsies, and resection of intra-uterine lesions. Thus preventing invasive and radical treatments and reducing post-operative morbidity.

However, the greatest advancements have been realized in cellular and molecular biology, in cytogenetics, and in proteomics. Indeed, the recent knowledge in the field of stem cells and their specific reprograming expand horizons to gene and cell therapy. A simple cell could be considered as a treatment by re-colonizing unhealthy tissues, thus preventing transplantation (1, 4, 5). The cryopreservation of ovarian tissue by vitrification before radiotherapy or chemotherapy in case of cancer gives a new hope to those couples touched by the disease.

The *in vitro* culture of pathological cells, disease modeling, and collection of withdrawn tissues pre-operatively allows researchers to understand etiophysiopathogeny, and natural evolution of the disease as in endometriotic tissues (2). These procedures will avoid long, expensive, and ethically questionable studies on animal models, thus allowing pharmacotesting. Therefore, this will help defining new disease classification and propose therapeutic algorithms.

Owing to progress in the field of infertility and assisted reproductive techniques (ART), i.e., intra cytoplasmic spermatozoid injection (ICSI) and vitrification of oocytes and embryos, increasing numbers of patients could be treated. *In vitro* endometrial culture leads to identification of reasons for treatment failure. Regarding pre-implantation genetic diagnosis (PGD), the progress in the knowledge of genetic mapping offers much hope to patients and couples suffering from family and hereditary diseases.

A better knowledge of human papilloma viruses (HPV) and the conception of vaccination against those HPV contribute in the reduction of the prevalence of cervical cancer, since HPV is involved in the genesis of cervical dysplasia and thus in that of cervical cancer. A radical treatment of cervical dysplasia (surgical or laser CO_2_ conization) prevents their transformation toward an invasive cervical cancer. The use of CO_2_ laser coupled with colposcopy allowed “see-and-treat” management of cervical, vaginal, vulvar, and perineal lesions related to HPV in an ambulatory way with a minimal morbidity. Eradication of cervical cancer during this century is possible and conceivable, only if national programs of information of gynecologists, family practitioners, as well as a program of HPV vaccination are settled.

In case of voiding dysfunction, stress urinary incontinence, overactive bladder, the innovations in the field of pressure sensors improved urodynamic measurements. A new clinical approach based on an itemized analysis of symptoms associated with progress of urodynamic tools allows a better therapeutic approach including both surgery and medical treatment.

MRI, computerized mammography, 3D ultrasound, stereotactic, and ultrasound guided biopsies of breast lesions eased and fastened the diagnosis of breast cancer, with less morbidity. All these imaging techniques contributed to a better management of gynecological cancers and thus improved survival rates in these patients. Sentinel axillary lymph node biopsy with intra-operative frozen section allows to ovoid the systematically axillary lymph node resection. Protocols such as “ONCOTYPE,” based on cellular phenotyping, predict the need for chemotherapy. As an example, Her-2–Neu is used as a marker of poor prognosis and indicator for the use of HERCEPTIN neoadjuvant treatment. Radiotherapy becomes more focused on the tumor site, thus avoiding overdoses of irradiation and reducing side effects. Hormonal therapy is possible due to immunochemistry analysis of tumor-estrogen and -progesterone receptors, thus offering a longer period without recurrence. In case of mixed ovarian tumors, immunohistochemistry allows an accurate diagnosis.

In the fields of contraception, a lot of progress has been achieved such as Levonorgestrel intra-uterine devices, progestogens of third and fourth generation allowing a decrease in ethinyl-estradiol dosage, progestogens implants, vaginal rings, and transdermal patches. So women have a very wide choice and contraception can be individually adapted.

Gynecology is in constant progress allowing an ambulatory management with less morbidity thus reduced health costs, an earlier diagnosis with a better adapted and personalized treatment leading to higher survival rates.

Obstetrics also knew considerable progresses. Since the twentieth century, prenatal diagnosis of congenital malformations and genetic diseases is made easier and earlier, a tribute of progresses in the fields of ultrasound, amniocentesis, and chorionic villi sampling (CVS), thus allowing an optimal management with less morbidity and less psychological disturbance in the respect of national legal frames. These great achievements could not be possible without progress in genetics and biology. Many markers are currently studied to screen earlier patients predisposed to develop preeclampsia during pregnancy or to predict premature rupture of membranes and labor, thus preventing wet lung syndrome. Progresses in neonatal resuscitation decreased perinatal morbidity and mortality. The detection of fetal DNA in the maternal blood with the hope of the complete genome fetal sequencing represents another breakthrough.

Henceforth, *in utero* fetal surgery as a vesico-amniotic shunting (a twin–twin transfusion syndrome with Laser ablation of vessels), the treatment of fetal bladder obstructions, an aortic or pulmonary valvuloplasty (opening the aortic or pulmonary fetal heart valves to allow blood flow) (6), an atrial septostomy (opening the inter-atrial septum of the fetal heart to allow unrestricted blood flow between the atriums), the surgical treatment of a congenital diaphragmatic hernia by a Balloon tracheal occlusion and the treatment of spina bifida with a closure of the malformation entered clinical practice.

Epidural anesthesia, the new standardized criteria of fetal heart rate analysis, the advent of the STAN, the analysis of scalp pH contributed to the improvement of perinatal prognosis. Techniques of embolization contributed to a less aggressive, but effective, treatment of post-partum hemorrhage, thus saving lives and sparing uteri.

With such a therapeutic arsenal, there should be no more fatality. However questions arise, among them “should we go back to a more physiological or natural practice of obstetrics?” or “why the rate of cesarean sections increased, and all this technology is available?” Even though we cannot compromise progresses, the question is to be debated.

In both gynecology and obstetrics, optimal training of the nurses, midwifes, and residents is essential. In these fields, specific simulators are ideal tools to start, maintain, and develop skills. Finally, internet and other media are obvious tools to enhance knowledge for patients, doctors, midwives, and nurses.

## Conflict of Interest Statement

The authors declare that the research was conducted in the absence of any commercial or financial relationships that could be construed as a potential conflict of interest.
